# The Role of Immune Dysregulation Markers in Cardiovascular Risk of People Living with HIV: Association Among Intima Media Changes, CD4/CD8 Ratio, and CD4+ Cell Count Nadir

**DOI:** 10.3390/v18030383

**Published:** 2026-03-18

**Authors:** Manuela Ceccarelli, Elena Delfina Ricci, Camilla Muccini, Laura Galli, Sergio Ferrara, Alessandra Tartaglia, Benedetto Maurizio Celesia, Elio Manzillo, Alessandra Guida, Giovanni Di Filippo, Rosa Basile, Antonella Castagna, Paolo Maggi

**Affiliations:** 1 Department of Medicine and Surgery, “Kore” University of Enna, 94100 Enna, Italy; manuela.ceccarelli@unikore.it; 2 Fondazione per lo Studio delle Infezioni e delle Allergopatie (ASIA) Onlus, 20900 Milan, Italy; ed.ricci@libero.it; 3 Unit of Infectious Diseases, Università Vita e Salute “San Raffaele”, 20132 Milan, Italy; 4 Unit of Infectious Diseases, Università degli Studi di Foggia, 71122 Foggia, Italy; 5 Unit of Infectious Diseases, Azienda Ospedaliera di Foggia, 71122 Foggia, Italy; 6 Unit of Infectious Diseases, “Nesima” Hospital, ARNAS “Garibaldi”, 95122 Catania, Italy; 7 UOC Malattie Infettive dell’Immunodepresso e Medicina di Genere, Ospedale “Cotugno”, AO dei Colli, 80131 Naples, Italy; 8 UOC Malattie Infettive dell’Immunodepresso e dell’Immigrazione, Ospedale “Cotugno”, AO dei Colli, 80131 Naples, Italy; 9 Unit of Infectious Diseases, Università degli Studi “Federico II” di Napoli, 80138 Naples, Italy; 10 Unit of Infectious Diseases, Grande Ospedale Metropolitano, 89133 Reggio Calabria, Italy

**Keywords:** cardiovascular risk, carotid vessels, HIV, intima–media thickness, plaques, CD4+, CD8+, ratio, nadir

## Abstract

HIV infection can promote persistent immune activation and endothelial dysfunction, contributing to atherosclerosis. Carotid intima–media thickness (cIMT) is an established marker of subclinical atherosclerosis. We evaluated the association between cIMT severity and two routinely available markers of immune dysregulation (CD4/CD8 ratio and nadir CD4+ cell count) in people living with HIV (PLWH). We conducted an Italian multicenter cross-sectional study including 1148 PLWH who underwent carotid color Doppler ultrasound. We classified cIMT as ≤0.9, 1.0–1.4, or >1.4 mm and analyzed these categories using multinomial logistic regression, reporting adjusted odds ratios (aORs) with 95% confidence intervals (CIs). We adjusted models for age, sex, BMI, HIV acquisition risk factor, hypertension, diabetes, dyslipidemia/statin use, triglycerides, integrase inhibitor use, and ART duration. cIMT was ≤0.9 mm in 615 (53.6%) participants, 1.0–1.4 mm in 379 (33.0%), and >1.4 mm in 154 (13.4%). Using nadir CD4+ ≥ 200 cells/µL and CD4/CD8 ≥ 1.0 as reference, PLWH with nadir CD4+ < 200 and CD4/CD8 ≥ 1.0 had higher odds of cIMT 1.0–1.4 mm (aOR 1.66, 95% CI 1.02–2.69) and >1.4 mm (aOR 3.45, 95% CI 1.68–7.07). In conclusion, CD4+ nadir and this combined pattern were associated with greater cIMT severity, supporting a role for immune dysregulation in subclinical atherosclerosis.

## 1. Introduction

HIV infection is associated with an increased risk of cardiovascular disease (CVD), even in the era of highly active antiretroviral therapy (ART) [1]. This is at least partly due to chronic inflammation induced by the virus. Studies have shown that HIV can lead to persistent immune activation and endothelial dysfunction, contributing to atherosclerosis and other cardiovascular events [2]. Additionally, some antiretroviral medications can have side effects that further increase cardiovascular risk, such as alterations in lipid metabolism [3].

CD4/CD8 ratio inversion and CD4+ cell count nadir are recognized as significant markers of chronic immune dysregulation in people living with HIV (PLWH). CD4/CD8 ratio inversion, characterized by a decrease in CD4+ lymphocytes and an increase in CD8+ lymphocytes, reflects a state of persistent immune activation and immunosenescence, contributing to an increased risk of non-AIDS-related comorbidities [4]. Similarly, nadir CD4+ count, representing the lowest point of CD4+ cell count during HIV infection, has been associated with subclinical atherosclerosis and cardiovascular risk in treated PLWH, independent of viral suppression achieved with ART [5,6]. These markers may provide clinically relevant insights into HIV-related immune dysregulation and help identify individuals at higher cardiovascular risk.

Carotid intima–media thickness (cIMT) and atheromatous plaques, detected by ultrasound, are early subclinical signs of atherosclerosis and independent predictors of cardiovascular events [7]. Previous studies have shown a correlation between cIMT and various traditional and non-traditional risk factors in HIV-positive individuals [5,8,9,10]. However, in this population, the role of chronic immune dysregulation markers, such as the CD4/CD8 ratio and nadir CD4+ cell count, in atherogenesis, remains incompletely understood.

In this cross-sectional study, we evaluated cIMT severity in a cohort of 1148 PLWH, analyzing its association with the CD4/CD8 ratio and nadir CD4+ cell count. The primary objective was to determine whether these markers of chronic immune dysregulation are independent predictors of cIMT severity, after adjusting for traditional cardiovascular risk factors. The results of this study may provide new insights into the mechanisms of atherogenesis in HIV-positive individuals and contribute to identifying more effective cardiovascular prevention strategies.

## 2. Materials and Methods

In this observational, retrospective, cross-sectional study we enrolled 1148 PLWH in the Archiprevaleat cohort. When multiple examinations were available for the same participant, only the most recent examination per participant was retained. Clinical and laboratory variables were retrieved from the visit corresponding to the ultrasound examination. The primary endpoint of the study was the evaluation of the association between intima media changes (cIMT severity), CD4/CD8 ratio inversion (<1.0), and CD4+ count nadir. Secondary endpoints were the evaluation of the association between cIMT and traditional risk factors for cardiovascular disease.

The Archiprevaleat cohort is described in a previous article [11]. Briefly, Archiprevaleat is a National Registry of color Doppler ultrasound, created to evaluate the characteristics of vascular lesions in a large sample of PLWH. The project currently involves nine Italian centers where the examination is carried out by specially trained clinicians. The Registry is based on an online platform aimed at collecting data on cIMT severity in patients routinely subjected to the examination. All PLWH had a Doppler scan of the supra-aortic vessels between 2009 and 2023. We evaluated the following parameters: IMT of common and internal carotid for both left and right sides; study method details have been previously published [11]. Thickness was recorded to the first decimal number. A cIMT > 0.9 mm was considered a pathological finding, while a cIMT > 1.4 mm was used to define a “severe cIMT” category, reflecting advanced carotid wall changes, rather than a morphologically defined plaque [12]. Atherosclerotic plaques, if present, were described. All images were photographed and archived. Although plaque presence was available in the dataset, it was not included in the current analysis, which was designed to focus on cIMT as a continuous/graded measure.

At each visit, we collected data regarding independent risk factors for CVD: total serum cholesterol, low-density lipoprotein (LDL) cholesterol, high-density lipoprotein (HDL) cholesterol, glycemia, triglycerides, and body mass index (BMI) and blood pressure (BP). Similarly, HIV viral load and CD4+ and CD8+ cell counts were recorded at each visit. CD4+ nadir was retrieved by each participating center from the local clinical records/electronic medical charts and/or local clinical databases and entered into the Archiprevaleat online registry. Nadir CD4 was defined as the lowest documented CD4+ cell count available in the clinical history prior to, or at the time of, the ultrasound examination. When historical laboratory data were incomplete, the value was recorded as missing and handled as described in the statistical analysis.

Hypertension was defined as office systolic BP of ≥140 mmHg and/or a diastolic BP of ≥90 mmHg, or receiving antihypertensive therapy at the time of the examination. Dyslipidemia refers to levels of one or more kinds of lipids in the blood (triglycerides > 200 mg/dL, total cholesterol > 240 mg/dL, LDL > 140 mg/dL) and/or the use of statins and other lipid-lowering drugs. Diagnosis of diabetes was based on standard international criteria [13].

The HIV viral load was assessed by real-time PCR with the lowest detection limit of 40 copies/mL. Lymphocyte subsets (CD4+, CD8+) were evaluated with flow cytofluorimetry using monoclonal antibodies and a fluorescence-activated cell-sorter scan (Becton Dickinson, Mountain View, CA, USA). Serum lipids and routine analyses were performed, applying standard procedures.

### Statistical Analysis

Continuous variables were described as means and standard deviation (SD) if normally distributed and as medians and interquartile range (IQR) if not normally distributed. To evaluate the distribution of continuous variables we used the Shapiro–Wilk test, and to assess homoscedasticity, we used the Levene test. Categorical variables were described as absolute frequency (n) and percentage (%). The univariate tests used to compare groups were the analysis of variance (ANOVA) with post hoc analysis, and the Pearson chi-squared test (or Fisher test or Mantel–Haenszel test, as appropriate), respectively.

We used multinomial logistic regression to estimate odds ratios (ORs) with 95% confidence intervals (CIs) to determine those belonging to higher cIMT categories (1.0–1.4 and >1.4 mm) compared with the reference group (cIMT ≤ 0.9 mm). To retain participants with incomplete covariate data, missing values were modelled as a separate category; however, this approach does not eliminate potential bias if data are not missing completely at random. In the multivariate equation, covariates were selected a priori based on clinical relevance, established cardiovascular risk factors; univariate associations (*p* < 0.05) were used to support model specification. To assess the robustness of our findings to the choice of CD4/CD8 categorization, we pre-specified a sensitivity analysis repeating the models with a CD4/CD8 cut-off of 0.5. All statistical analyses were performed using SAS 9.4 for Windows (SAS Institute, Cary, NC, USA).

## 3. Results

Since March 2009, 1148 PLWH underwent cIMT evaluation. The median age was 51 years (IQR 44–57), and the proportion of women was 20.7%. HIV transmission route was mainly sexual (60.3%), while history of previous injecting drug use (IDU) was 13.2%. Hypertension was present in 33.4% and diabetes in 8.8% of the sample. Dyslipidemia was the most prevalent comorbidity, present in 58.7%. Statin treatment was reported in 26.3% of the sample.

cIMT was ≤0.9 mm in 615 (53.6%) PLWH, between 1.0 and 1.4 mm in 379 (33.0%), and >1.4 mm in 154 (13.4%). The characteristics of our sample according to this classification are reported in Table 1.

As expected, the groups were different in terms of age, sex, BMI, prevalence of hypertension, diabetes, dyslipidemia, and median values of triglycerides. Regarding HIV-related variables, current integrase strand transfer inhibitor (INSTI)-based regimens, ART duration, and nadir CD4+ < 200 cell/µL were more frequent in people with cIMT > 1.4 mm.

In Table 2, ORs from the univariate and multivariate analyses are reported. Covariates included in the multivariable models were selected a priori based on clinical relevance, prior literature, and univariate associations: age, sex, BMI, risk factor for HIV acquisition, hypertension (treated and untreated), diabetes, dyslipidemia and use of statins, blood triglycerides, INSTI-based regimen at the time of examination, and ART duration. In two different equations, we included nadir CD4+ (<200 or ≥200 cell/µL) and the current CD4/CD8 ratio (<1.0 or ≥1.0) as separate variables, or a new variable derived from their combination.

The multivariate model included all variables with *p* < 0.05 at the univariate analysis: age class, sex, BMI, risk factor for HIV acquisition, high blood pressure (and treatment), diabetes, statin use, INSTI-including regimen, and blood triglycerides. Alternatively, models included nadir CD4+ level and CD4/CD8 ratio, or the combination of these two variables.

In the first model, a nadir CD4+ level < 200 cell/µL was linked to higher odds of cIMT between 1.0 and 1.4 mm (aOR 1.37, 95% CI 1.01–1.84) and for cIMT > 1.4 mm (aOR 2.26, 95% CI 1.43–3.59), whereas, using CD4/CD8 ≥ 1.0 as the reference, a CD4/CD8 ratio < 1.0 was associated with lower odds of both cIMT 1.0–1.4 mm (aOR 0.71, 95% CI 0.53–0.95) and cIMT > 1.4 mm (aOR 0.58, 95% CI 0.37–0.89). When including the variable derived from the combination, using PLWH with nadir CD4+ ≥ 200 cell/µL + CD4/CD8 ratio ≥ 1.0 as the reference, we found no significant difference with other groups (nadir CD4+ < 200 cell/µL + CD4/CD8 ratio < 1.0 and nadir CD4+ ≥ 200 cell/µL + CD4/CD8 ratio < 1.0), except for PLWH with nadir CD4+ < 200 cell/µL and CD4/CD8 ratio ≥ 1.0. They showed higher odds of both cIMT between 1.0 and 1.4 mm (aOR 1.66, 95% CI 1.02–2.69) and cIMT > 1.4 mm (aOR 3.45, 95% CI 1.68–7.07).

To further investigate the relationship between nadir CD4+ and immune reconstitution, we re-ran the same multivariate model using a different cutoff for the CD4/CD8 ratio. PLWH with CD4/CD8 ratio ≥ 0.5 showed a tendency towards lower odds of both cIMT 1.0–1.4 (aOR 0.82, 95% CI 0.57–1.17) and >1.4 mm (aOR 0.78, 95% CI 0.47–1.31), but the estimates were higher than using the 1.0 cutoff (Table 2), and statistically non-significant. Regarding the combination of nadir CD4+ and CD4/CD8 ratio, we observed similar results, with the highest aOR for people with nadir CD4+ < 200 cells/µL and CD4/CD8 ratio ≥ 0.5 (aOR 2.08, 95% CI 1.27–3.40), which is still lower than using the 1.0 cutoff. In the combined-variable model, participants with nadir CD4+ ≥ 200 cells/µL and CD4/CD8 ratio < 1.0 showed point estimates below 1.0 for both cIMT categories (aOR 0.77, 95% CI 0.51–1.17 for cIMT 1.0–1.4 mm; aOR 0.83, 95% CI 0.42–1.66 for cIMT > 1.4 mm), although these associations were not statistically significant. All the remaining estimates were statistically non-significant (Table S1).

## 4. Discussion

Residual inflammation is a major determinant of CVD risk in PLWH [14,15,16]. Evidence regarding the incremental value of circulating inflammatory biomarkers for CVD risk prediction is mixed, and their added discriminative performance remains uncertain [14,15,16]. However, some studies suggested that CD4/CD8 ratio and CD8+ count could predict severe non-AIDS related events [17,18].

In the present study, as expected, we found a role of traditional risk factors for CVD, such as age, sex, BMI, hypertension, diabetes, and median values of triglycerides, in the onset of cIMT ≥ 1.00–1.40 mm and cIMT > 1.40 mm.

Regarding HIV-related factors we found a significant role for a longer period of ART treatment in the presence of a cIMT > 1.4 mm. This could be due to exposure to older antiretrovirals at risk for cardiometabolic toxicity, such as boosted protease inhibitors or abacavir [3]. Also, current INSTI-based regimens were associated with cIMT > 1.4 mm. Other studies found an association between the use of INSTI and diabetes [19], hypertension [20], or cardiovascular events [21], although we cannot exclude a channeling bias since INSTI-based regimens are generally perceived as cardiovascular-friendly antiretroviral treatments.

Regarding immune dysregulation markers, we found that nadir CD4+ < 200 cell/µL was significantly associated with cIMT > 1.4 mm. Univariate and multivariate analyses confirmed that a nadir CD4+ level < 200 cell/µL was associated with higher odds of severe cIMT, supporting an association between profound historical immunodeficiency and greater cIMT burden, consistent with long-term immune perturbations.

We observed an unexpected association whereby a CD4/CD8 ratio < 1.0 was associated with lower odds of increased cIMT compared with CD4/CD8 ≥ 1.0. Because this is a cross-sectional analysis, we cannot infer directionality or causality; therefore, this finding should be viewed as hypothesis-generating rather than mechanistic. Notably, the signal was driven mainly by participants with a history of advanced immunodeficiency (CD4 nadir < 200 cells/µL) who later achieved a CD4/CD8 ratio ≥ 1.0. In the combined-variable model (reference: nadir CD4 ≥ 200 cells/µL and CD4/CD8 ≥ 1.0), this subgroup showed the highest odds of both cIMT 1.0–1.4 mm and cIMT > 1.4 mm (aOR 1.67 and 3.40, respectively), while most other nadir–ratio strata were not significant. This pattern suggests that, in some individuals, apparent immunologic “normalization” of CD4/CD8 after profound past CD4 depletion may identify a subgroup with distinct immune trajectories potentially relevant to vascular remodeling. Importantly, this should not be interpreted as evidence that CD4/CD8 < 1.0 is biologically protective; rather, the finding highlights the need for replication and for longitudinal studies incorporating inflammatory biomarkers and clinical endpoints. Given that CD4/CD8 < 1.0 is traditionally viewed as a marker of immune dysregulation, the apparent “protective” direction likely reflects subgroup structure or residual confounding rather than a causal protective effect.

Moreover, this is in contrast with other evidence suggesting that CD4/CD8 ratio inversion is associated with a state of persistent immune activation and an increased risk of non-AIDS-related comorbidities [4]. However, we hypothesized that a low CD4+ cell nadir, associated with a sharp immune reconstitution, could determine higher odds of chronic inflammation, as we observed in a previous study [22], where we described that patients experiencing a more rapid immune reconstitution developed a significantly higher number of subclinical vascular lesions. To corroborate this hypothesis, in two different models, we included nadir CD4+ (<200 or ≥200 cell/µL) and the current CD4/CD8 ratio (<1.0 or ≥1.0) as separate variables, or a new variable derived from their combination. In the latter model, PLWH with nadir CD4+ < 200 cell/µL and current CD4/CD8 ratio ≥ 1.0 were at higher odds of both cIMT between 1.0 and 1.4 mm and cIMT > 1.4 mm. Biological plausibility for this interpretation also comes from biomarker-based phenotyping work conducted in an independent cohort of PLWH: inflammatory clustering analyses identified a “gut-T-cell-inflamed” phenotype characterized by markers of gut epithelial dysfunction, T-cell differentiation/regulation, and systemic inflammation, and this phenotype was associated with prevalent CVD in adjusted analyses [23]. In a related metabolomics analysis of the same cohort presented at CROI 2025 [24] (conference poster, non-peer-reviewed), the highest-risk inflammatory cluster (defined by higher T-cell inflammation and gut endothelial dysfunction, previously linked to markedly higher CVD prevalence) also showed a higher CD4/CD8 ratio distribution (median 1.04) and a coordinated downregulation of anti-inflammatory kynurenine-pathway metabolites in the kynurenic-acid branch, consistent with a pro-inflammatory milieu despite apparent immunologic recovery.

Our study has some limitations. Missing covariates were modelled as a separate category; if data were not missing completely at random, residual bias may persist. In addition, inflammatory biomarkers were not measured in the present study, and unmeasured confounding remains possible. Moreover, key cardiovascular confounders were not available in our dataset (including smoking status, family history of CVD, HCV/other co-infections, renal function, and lifestyle factors such as physical activity and diet), and residual confounding cannot be excluded. Additionally, because the cohort was overwhelmingly Caucasian, ethnicity could not be robustly evaluated in multivariable analyses and generalizability to other ethnic groups is limited. Duration of HIV infection (e.g., time since diagnosis/seroconversion) was not consistently available and could not be directly adjusted for; ART duration was included as a partial proxy, but residual confounding related to infection duration cannot be excluded. Longitudinal trajectories of CD4/CD8 ratio were not available in a standardized manner for the full cohort; therefore, we could not evaluate changes over time. This should be addressed in future longitudinal studies. Finally, we did not account for potential center- or calendar year-related heterogeneity (e.g., ultrasound protocols, operator variability, or changes in clinical practice over time), which may have affected the estimates. Although plaque presence was recorded in the registry, the present analysis focused on cIMT severity; analyses incorporating plaque presence are planned for future work.

## 5. Conclusions

We observed that CD4+ cell count nadir and the combination of nadir CD4+ < 200 cell/µL and CD4/CD8 ratio ≥ 1.0 are associated with higher frequency of cIMT alterations. These findings support the hypothesis that immune dysregulation related to prior immunodeficiency and subsequent immune reconstitution may be linked to subclinical atherosclerosis.

## Figures and Tables

**Table 1:** Characteristics of 1148 people with HIV enrolled in the Archiprevaleat Project.

	cIMT ≤ 0.9 mm N = 615 (53.6%)	cIMT 1.0–1.4 mm N = 379 (33.0%)	cIMT > 1.4 mm N = 154 (13.4%)	*p*
**Age, years, median (IQR)**	48 (41–54)	52 (48–59)	57 (53–64)	<0.0001
**Age class, years, *n* (%)**				<0.0001
**<40**	134 (21.8)	42 (11.1)	2 (1.3)	
**40–49**	219 (35.6)	84 (22.2)	14 (9.1)	
**50–59**	186 (30.2)	162 (42.7)	77 (50.0)	
**60–69**	64 (10.4)	73 (19.3)	45 (29.2)	
**≥70**	12 (2.0)	18 (4.8)	16 (10.4)	
**Male, *n* (%)**	465 (75.6)	313 (82.6)	132 (85.7)	0.003
**Caucasian**	605 (98.4)	362 (95.5)	149 (96.8)	0.03
**BMI > 25.0 (kg/m^2^), *n* (%)**	220 (35.8)	169 (44.6)	77 (50.0)	0.0008
**Missing**	67 (10.9)	51 (13.5)	13 (8.4)	
**Risk factor for HIV acquisition, *n* (%)**				0.05
**IDU**	72 (11.7)	53 (14.0)	27 (17.5)	
**Sexual**	391 (63.6)	215 (56.7)	86 (55.8)	
**Transfusion**	7 (1.1)	2 (0.5)	4 (2.6)	
**Other/unknown**	145 (23.6)	109 (28.8)	37 (24.0)	
**Hypertension, *n* (%)**				<0.0001
**Treated, *n* (%)**	113 (18.4)	120 (31.7)	67 (43.5)	
**Untreated, *n* (%)**	42 (6.8)	21 (5.5)	20 (13.0)	
**Diabetes, *n* (%)**	32 (5.2)	36 (9.5)	33 (21.4)	<0.0001
Dyslipidemia *, *n* (%)	317 (51.5)	241 (63.6)	116 (75.3)	<0.0001
**On statins, *n* (%)**	133 (21.6)	103 (27.2)	66 (42.9)	<0.0001
**Total cholesterol, mg/dL, median (IQR)**	184 (159–214)	191 (163–222)	193 (163–219)	0.08
**LDL-cholesterol, mg/dL, median (IQR)**	114 (94–140)	118 (95–147)	114 (88–140)	0.29
**Triglycerides, mg/dL, median (IQR)**	99 (65–146)	105 (74–160)	130 (90–192)	<0.0001
**Current PI, *n* (%)**	237 (38.5)	127 (33.5)	49 (31.8)	0.14
**Current NNRTI, *n* (%)**	182 (29.6)	110 (29.0)	50 (32.5)	0.72
**Current INSTI, *n* (%)**	172 (28.0)	134 (35.4)	57 (37.0)	0.02
**Years of ART, median (IQR)**	11.3 (4.4–17.4)	13.6 (6.0–19.4)	16.8 (10.2–22.8)	<0.0001
**CD4+, cell/µL, median (IQR)**	686 (496–906)	674 (485–962)	668 (429–881)	0.47
**CD8+, cell/µL, median (IQR)**	860 (630–1172)	804 (585–1116)	834 (592–1153)	0.09
**Nadir CD4+ < 200 cell/µL, *n* (%)**	296 (48.1)	198 (52.2)	96 (62.3)	0.0003
**Missing**	26 (4.2)	23 (6.1)	14 (9.1)	
**CD4/CD8 ratio, median (IQR)**	0.82 (0.51–1.17)	0.84 (0.57–1.20)	0.81 (0.50–1.25)	0.33
**CD4/CD8 ratio ≥ 1.0, *n* (%)**	218 (35.4)	149 (39.3)	62 (40.3)	0.34
**Combined nadir CD4+ and CD4/CD8 ratio, *n* (%)**				0.0006
**Nadir CD4+ < 200 cell/µL + CD4/CD8 ratio < 1**	226 (36.8)	139 (36.7)	63 (40.9)	
**Nadir CD4+ < 200 cell/µL + CD4/CD8 ratio ≥ 1**	70 (11.4)	59 (15.6)	33 (21.4)	
**Nadir CD4+ ≥ 200 cell/µL + CD4/CD8 ratio < 1**	162 (26.3)	80 (21.1)	23 (14.9)	
**Nadir CD4+ ≥ 200 cell/µL + CD4/CD8 ratio ≥ 1**	131 (21.3)	78 (20.6)	21 (13.6)	
**Missing**	26 (4.2)	23 (6.1)	14 (9.1)	

ART: antiretroviral treatment; BMI: body mass index; IDU: intravenous drug use; cIMT: carotid intima–media thickness; INSTI: integrase strand transfer inhibitor; IQR: interquartile range; NNRTI: non-nucleoside reverse transcriptase inhibitor; PI: protease inhibitor. * on lipid-lowering treatment, or total cholesterol > 240 mg/dL, or triglycerides > 200 mg/dL, or LDL-cholesterol > 140 mg/dL.

**Table 2:** Univariate and multivariate analysis for the risk of pathological cIMT 1.0–1.4 and >1.4 (reference category IMT ≤ 0.9).

	Odds Ratio (95% Confidence Interval)		Adjusted Odds Ratio (95% Confidence Interval)	
	cIMT 1.0–1.4 mm	cIMT > 1.4 mm	cIMT 1.0–1.4 mm	cIMT > 1.4 mm
**Age class, years, ref. < 50**	1.00	-	1.00	-
50–59	2.44 (1.82–3.27)	9.13 (5.18–16.10)	2.13 (1.54–2.95)	6.55 (3.510–12.23)
60–69	3.20 (2.16–4.73)	15.51 (8.26–29.11)	2.68 (1.73–4.15)	9.66 (4.77–19.55)
≥70	4.20 (1.97–8.97)	29.42 (11.95–72.40)	4.29 (1.89–9.74)	21.87 (7.880–60.69)
**Sex, ref. M**	1.00	-	1.00	-
F	0.65 (0.47–0.90)	0.52 (0.32–0.84)	0.71 (0.50–1.01)	0.50 (0.28–0.89)
**BMI, ref. ≤ 25.0**	1.00	-	1.00	-
>25.0	1.58 (1.20–2.09)	1.79 (1.24–2.70)	1.50 (1.11–2.04)	1.96 (1.26–3.05)
Missing	1.57 (1.04–2.37)	1.00 (0.52–1.91)	1.54 (0.98–2.52)	1.22 (0.58–2.55)
**Risk factor for HIV acquisition, ref. sexual**	1.00	-	1.00	-
IDU	1.34 (0.90–1.98)	1.70 (1.03–2.81)	1.08 (0.69–1.68)	1.23 (0.67–2.26)
Transfusion	0.52 (0.11–2.52)	2.60 (0.74–9.07)	0.16 (0.02–1.34)	1.21 (0.26–5.50)
Other/unknown	1.37 (1.01–1.84)	1.16 (0.76–1.78)	1.14 (0.82–1.58)	0.92 (0.56–1.52)
**Hypertension, ref. N**	1.00	-	1.00	-
Treated, *n* (%)	2.05 (1.52–2.77)	4.07 (2.74–6.05)	1.44 (1.02–2.03)	1.61 (0.99–2.59)
Untreated, *n* (%)	0.97 (0.56–1.67)	3.27 (1.81–5.90)	0.86 (0.48–1.56)	2.04 (1.00–4.14)
**Diabetes, ref. N**	1.00	-	1.00	-
Y	1.91 (1.17–3.14)	4.97 (2.94–8.39)	1.17 (0.68–2.00)	1.99 (1.08–3.68)
Dyslipidemia *, ref. N	1.00	-	1.00	-
Y	1.64 (1.26–2.13)	2.87 (1.92–4.28)	1.18 (0.88–2.59)	1.66 (1.03–2.66)
**Statin treatment, ref. no dyslipidemia**	1.00	-	1.00	-
On statins	1.67 (1.21–2.32)	3.89 (2.48–6.09)	1.04 (0.71–1.52)	1.82 (1.06–3.14)
Not on statins	1.62 (1.20–2.18)	2.13 (1.34–3.38)	1.28 (0.92–1.78)	1.49 (0.88–2.54)
**Total cholesterol, by 10 mg/dL**	1.03 (1.00–1.06)	1.03 (0.98–1.07)	-	-
**LDL-cholesterol, by 10 mg/dL**	1.01 (0.98–1.04)	0.98 (0.94–1.03)	-	-
**Triglycerides, by 10 mg/dL**	1.02 (1.01–1.03)	1.03 (1.02–1.05)	1.16 (1.01–1.34)	1.32 (1.10–1.57)
**Current PI, ref. N**	1.00	-	-	-
Y	0.80 (0.62–1.05)	0.74 (0.51–1.08)	-	-
**Current NNRTI, ref. N**	1.00	-	-	-
Y	0.97 (0.73–1.29)	1.14 (0.78–1.67)	-	-
**Current INSTI, ref. N**	1.00	-	1.00	-
Y	1.41 (1.07–1.85)	1.51 (1.04–2.19)	1.33 (0.98–1.80)	1.16 (0.74–1.82)
**Years of ART, by 1**	1.03 (1.02–1.05)	1.08 (1.06–1.11)	1.03 (1.01–1.05)	1.05 (1.02–1.08)
**CD4+, cell/µL, by 100**	1.01 (0.97–1.04)	0.98 (0.93–1.03)	-	-
**CD8+, cell/µL, by 100**	0.98 (0.95–1.00)	0.98 (0.95–1.02)	-	-
**Nadir CD4+, ref. ≥ 200 cell/µL**	1.00	-	1.00	-
<200 cell/µL	1.24 (0.95–1.62)	2.16 (1.46–3.20)	1.37 (1.01–1.84)	2.26 (1.43–3.59)
Missing	1.64 (0.91–2.67)	3.59 (1.74–7.39)	1.59 (0.83–3.05)	3.62 (1.49–8.83)
**CD4/CD8 ratio, by 0.1**	1.02 (0.99–1.04)	1.01 (0.98–1.04)	-	-
**CD4/CD8 ratio, ref. ≥ 1.0**	1.00	-	1.00	-
<1.0	0.85 (0.65–1.10)	0.82 (0.57–1.17)	0.71 (0.53–0.95)	0.58 (0.37–0.89)
*Alternative analysis*				
**Nadir CD4+ ≥ 200 cell/µL + CD4/CD8 ratio ≥ 1, ref.**	1.00	-	1.00	-
Nadir CD4+ < 200 cell/µL + CD4/CD8 ratio < 1	1.03 (0.73–1.47)	1.74 (1.02–2.98)	0.96 (0.65–1.41)	1.43 (0.78–2.65)
Nadir CD4+ < 200 cell/µL + CD4/CD8 ratio ≥ 1	1.42 (0.91–2.21)	2.94 (1.58–5.46)	1.66 (1.02–2.69)	3.45 (1.68–7.07)
Nadir CD4+ ≥ 200 cell/µL + CD4/CD8 ratio < 1	0.83 (0.56–1.22)	0.89 (0.47–1.67)	0.77 (0.51–1.17)	0.83 (0.42–1.66)

ART: antiretroviral treatment; BMI: body mass index; IDU: intravenous drug use; cIMT: carotid intima–media thickness; INSTI: integrase strand transfer inhibitor; NNRTI: non-nucleoside reverse transcriptase inhibitor; PI: protease inhibitor. * on lipid-lowering treatment, or total cholesterol > 240 mg/dL, or triglycerides > 200 mg/dL, or LDL-cholesterol > 140 mg/dL.

## Data Availability

The raw data supporting the conclusions of this article will be made available by the authors on request.

## Supplementary Materials

The following supporting information can be downloaded at: https://www.mdpi.com/article/10.3390/v18030383/s1, Table S1: Univariate and multivariate analysis for the risk of pathological cIMT 1.0–1.4 and >1.4 (reference category IMT ≤ 0.9).

## Abbreviations

The following abbreviations are used in this manuscript:

CVD	Cardiovascular disease
ART	Antiretroviral therapy
PLWH	People living with HIV
cIMT	Carotid intima–media thickness
LDL	Low-density lipoprotein cholesterol
HDL	High-density lipoprotein cholesterol
BMI	Body mass index
BP	Blood pressure
SD	Standard deviation
IQR	Interquartile range
ANOVA	Analysis of variance
CI	Confidence interval
IDU	Injecting drug use
INSTI	Integrase strand transfer inhibitor
NNRTI	Non-nucleoside reverse transcriptase inhibitor
NRTI	Nucleoside reverse transcriptase inhibitor
PI	Protease inhibitor
