# Supplementary material for: The Role of Immune Dysregulation Markers in Cardiovascular Risk of People Living with HIV: Association Among Intima Media Changes, CD4/CD8 Ratio, and CD4+ Cell Count Nadir
Source: Viruses. 2026 Mar 18;18(3):383. doi: 10.3390/v18030383 (PMC13030327; doi:10.3390/v18030383)
Supplement: Supplementary file 1 [file viruses-18-00383-s001.zip › viruses-4191752-supplementary.pdf]

Table S1. Univariate and multivariate analysis for the risk of pathological IMT 1.0-1.4 and >1.4 (reference category IMT≤0.9).

	Odds Ratio (95% confidence interval)		Adjusted Odds Ratio (95% confidence interval)	
	**cIMT 1.0-1.4 mm**	**cIMT >1.4 mm**	**cIMT 1.0-1.4 mm**	**cIMT >1.4 mm**
**CD4+/CD8+ ratio, ref. ≥0.5**	1.00	-	1.00	-
<0.5	0.87 (0.63-1.20)	1.18 (0.78-1.78)	0.82 (0.57-1.17)	0.78 (0.47-1.31)
**Nadir CD4+ ≥200 cell/µL + CD4+/CD8+ ratio ≥0.5, ref.**	1.00	-	1.00	-
Nadir CD4+ <200 cell/µL + CD4+/CD8+ ratio <0.5	1.06 (0.53-1.56)	1.97 (1.16-3.36)	1.12 (0.74-1.71)	1.67 (0.89-3.15)
Nadir CD4+ <200 cell/µL + CD4+/CD8+ ratio ≥0.5	1.23 (0.92-1.66)	2.19 (1.42-3.37)	1.26 (0.91-1.74)	2.08 (1.27-3.40)
Nadir CD4+ ≥200 cell/µL + CD4+/CD8+ ratio <0.5	0.55 (0.26-1.15)	0.82 (0.27-2.43)	0.53 (0.23-1.21)	0.64 (0.20-2.11)
	**cIMT≤0.9 mm**	**cIMT 1.0-1.4 mm**	**cIMT >1.4 mm**	
	**N=615 (53.4%)**	**N=379 (33.0%)**	**N=154 (13.4%)**	**P**
**CD4+/CD8+ ratio ≥0.5, n (%)**	481 (78.2%)	305 (80.5%)	116 (75.3%)	0.40
**Combined Nadir CD4+ and CD4+/CD8+ ratio, n (%)**				
Nadir CD4+ <200 cell/µL + CD4+/CD8+ ratio <0.5	96 (15.6%)	58 (15.3%)	29 (18.8%)	
Nadir CD4+ <200 cell/µL + CD4+/CD8+ ratio ≥0.5	200 (32.5%)	140 (36.9%)	67 (43.5%)	
Nadir CD4+ ≥200 cell/µL + CD4+/CD8+ ratio <0.5	32 (5.2%)	10 (2.6%)	4 (2.6%)	
Nadir CD4+ ≥200 cell/µL + CD4+/CD8+ ratio ≥0.5	261 (42.4%)	148 (39.0%)	40 (26.0)	
missing	26 (3.2)	23 (6.1)	14 (9.1)	0.002

The multivariate model included all variables with p<0.05 at the univariate analysis: age class, sex, ethnicity, BMI, risk factor for HIV acquisition, high blood pressure (and treatment), diabetes, statin use, INSTI-including regimen, blood triglycerides. Models included nadir CD4 level and CD4/CD8 ratio, or the combination of these two variables, alternatively.
